# Postmortem Computed Tomography Imaging in the Investigation of Nontraumatic Death in Infants and Children

**DOI:** 10.1155/2013/327903

**Published:** 2013-09-04

**Authors:** Yukihiro Noda, Ken Yoshimura, Shoji Tsuji, Atsushi Ohashi, Hirohide Kawasaki, Kazunari Kaneko, Shigeki Ikeda, Hiroaki Kurokawa, Noboru Tanigawa

**Affiliations:** ^1^Department of Pediatrics, Kansai Medical University, 2-5-1 Shin-machi, Hirakata-shi, Osaka 573 1010, Japan; ^2^Department of Radiology, Kansai Medical University, Osaka 573 1010, Japan

## Abstract

*Objective*. To determine the accuracy of postmortem computed tomography (PMCT) for the assessment of causes in nontraumatic deaths in children. *Study Design*. We enrolled cases of nontraumatic deaths of infants and children who underwent PMCT at a single center. The presumed cause of death determined by PMCT was prospectively compared with the clinical and pathological diagnoses of deaths. *Results*. Thirty-eight cases were enrolled for analysis. Among them, seven cases also underwent conventional medical autopsy. PMCT revealed an identifiable cause of death in accordance with the clinical diagnosis of death in 16 cases of the 38 cases (the concordance rate was 42%) and in accordance with the autopsy cause of death in four of the seven autopsy cases (the concordance rate was 57%). Among eight cases with unknown cause of death by clinical diagnosis, four cases (50%) were identified with cardiac tamponade as a cause of death (one case) and intracranial hemorrhage suggesting abuse (3 cases). *Conclusions*. PMCT seems to be a promising technique that might serve as a substitute for conventional medical autopsy and give us the complementary information to clinical diagnoses particularly in cases of child abuse. Larger multicenter trials are worthwhile to validate the general feasibility of PMCT.

## 1. Introduction

Autopsy is considered the reference standard for postmortem evaluations held to identify the cause of death while the decline of autopsies among adults in most developed countries in the latter half of the 20th century and beyond is well established [1].

In children with unexpected death, a systematic conventional autopsy, with macroscopic and histological investigations, is usually offered to the parents of a child or infant who died as parental informed consent is required for an autopsy except in the case of forensic investigation. However, a global trend of declining pediatric autopsy rates has been reported as well as in adults because of the emotional, cultural, or religious reasons for refusing autopsy [2, 3]. Failure to obtain the parental informed consent for a pediatric autopsy also occurs frequently in Japan, mostly for emotional reasons given by the parents. Accordingly, the rate of autopsy in children in Japan is as low as 3% [4], which is extremely lower than that of western countries [1].

Several studies have reported the use of postmortem imaging in order to identify the cause of death in adults [5–8]. For example, Le Blanc-Louvry et al. suggested that the concordance between postmortem computed tomography (PMCT) and autopsy is almost perfect in determining the cause of death, and PMCT could be considered as effective as a forensic standard autopsy in determining the cause of death in certain traumatic events [7]; Roberts et al. found that PMCT was even more accurate imaging technique than postmortem MRI for determining the cause of death compared to traditional autopsy [5]. The reason for the usability of PMCT is as follows: the major discrepancy rate compared with autopsy was significantly higher for MRI than for PMCT; CT provides better spatial resolution than MRI and is effective for showing fractures and hemorrhages; CT has important practical advantages, being more widely available, less expensive, and quicker to be done than MRI [5].

On the contrary, there has been little focus on postmortem imaging specifically for pediatric populations. In the present study, we evaluated the accuracy of PMCT in the investigation of cause of nontraumatic death in infants and children at a single center basis.

## 2. Materials and Methods

### 2.1. Study Design

We studied the nontraumatic deaths of infants and children that occurred at a single center. The presumed cause of death obtained by the PMCT was prospectively compared with the clinical and pathological diagnoses of the cause of death.

### 2.2. Study Subjects

The study subjects were infants and children less than 12 years of age who died or were dead on arrival at Kansai Medical University Hirakata Hospital, Osaka, Japan, between January 2008 and March 2013. Written parental informed consent for PMCT imaging was obtained for the family of each patient enrolled in this study. The exclusion criteria were failure to obtain parental informed consent for PMCT imaging.

### 2.3. PMCT Technique

All PMCT images were acquired on a four-slice multidetector scanner (*Asteion Super4*; Toshiba Medical Systems Corp., Ohtawara, Tochigi, Japan). Contiguous axial images were obtained from the vertex to the pelvis with 3–8 mm slice thickness. The PMCT was performed with the cadaver in the natural supine position, with arms adjacent to the body. No contrast medium was administered.

### 2.4. Conventional Medical Autopsy Technique

An autopsy was performed for each subject within 24 h of death or dead on arrival by two pathologists after PMCT imaging. At the time of the autopsy, the pathologists were informed of the subject's clinical history, whereas they were blinded to the findings of the PMCT.

### 2.5. Radiologic Image Interpretation

The PMCT was interpreted by two radiologists (Shigeki Ikeda, Hiroaki Kurokawa), blind to the autopsy findings. They interpreted the PMCT images together for every case. Each of the radiologists had no previous experience of postmortem imaging at the time. The radiologists were informed of the clinical features in relation to the subject's medical history and previous clinical problems at the time of the image interpretation, whereas they were blinded to the pathological diagnoses of the deaths. Final image interpretation was reached in consensus. The presumed cause of death was established when fatal findings were detected on the PMCT images.

### 2.6. Data Collection and Analysis

Demographic data including the subjects' ages and gender and clinical data were collected. In addition, the causes of death were compared between the clinical diagnoses and the PMCT findings and also between the PMCT findings and the autopsy results.

## 3. Results

### 3.1. Study Population

**Figure 1:**
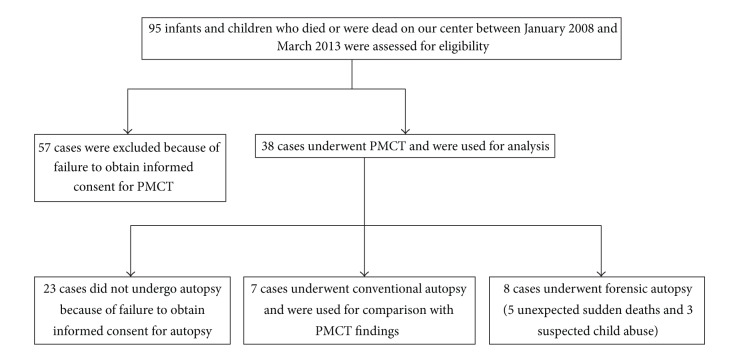
Study protocol. PMCT: postmortem computed tomography.

As demonstrated in Figure 1, ninety-five cases were eligible for the study. Among them, we excluded 57 cases because of failure to obtain informed consent for PMCT imaging, leaving 38 cases (40%) for analysis, 30 boys and 8 girls, age range from 0.0 year to 12.0 years (median age: 0.2 years). Seven of the 95 eligible cases (7%) also underwent a conventional medical autopsy after PMCT imaging as parental informed consent could be obtained. A forensic investigation was also performed in 8 cases of which findings were not disclosed to us by legal regulation.

PMCT imaging was performed very soon after the subject's death; that is, the interval time between certification of death and PMCT acquisition ranged from 0.3 h to 10.1 h (median interval time: 1.8 h). 

### 3.2. Causes of Death

**Figure 2:**
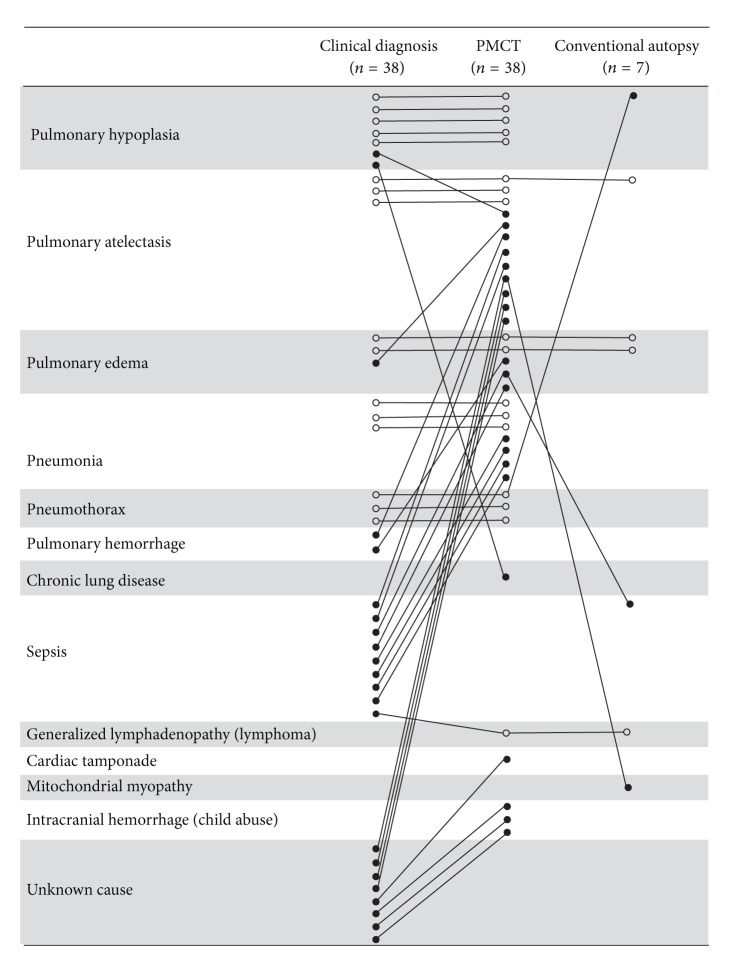
Concordance between postmortem examinations. Causes of death were compared between clinical diagnoses, PMCT, and conventional autopsy. Concordance between postmortem examinations, open circles; discordance between postmortem examinations, filled circles. PMCT: postmortem computed tomography.

**Figure 3:**
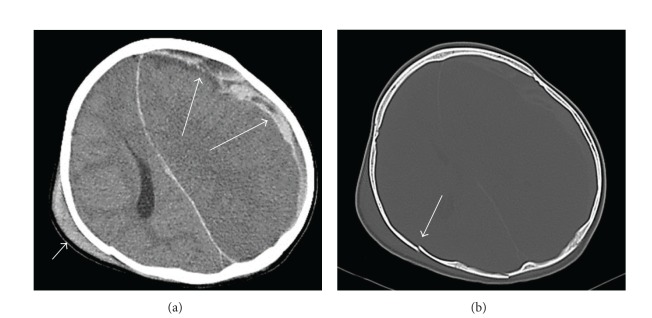
PMCT images of a 1-year-old boy with cardiopulmonary arrest. (a) Axial cranial PMCT image indicating subdural hemorrhage (long arrows) with subcutaneous hematoma (short arrow); (b) axial cranial PMCT image denoting occipital skull fracture (arrow). PMCT: postmortem computed tomography.

As shown in Figure 2, the causes of death could be established by clinical diagnosis only in 30 cases (79%) and by PMCT in all 38 cases (100%): the causes of death at PMCT were found to be pulmonary atelectasis (*n* = 12: 31.6%), pulmonary hypoplasia (*n* = 5: 13.2%), pneumonia (*n* = 7: 18.4%), pulmonary edema (*n* = 5: 13.2%), pneumothorax (*n* = 3: 7.9%), intracranial hemorrhage (*n* = 3: 7.9%), chronic lung disease (*n* = 1: 2.6%), cardiac tamponade (*n* = 1: 2.6%), and generalized lymphadenopathy (*n* = 1: 2.6%). PMCT revealed an identifiable cause of death in accordance with the clinical diagnosis of death in 16 of the 38 cases resulting in the concordance rate of 42%. PMCT also revealed an identifiable cause of death in accordance with the cause of death assessed by conventional autopsy in four of the seven cases resulting in the concordance rate of 57%, while in only three among them the causes of death by clinical diagnosis accord with those of conventional autopsy (concordance rate: 43%). It is of note that among eight cases in which the clinical diagnosis-based cause of death was “unknown,” four cases (50%) were diagnosed by PMCT: there was one case in which PMCT revealed cardiac tamponade as the cause of death, and there were three cases in which PMCT revealed intracranial hemorrhage as the cause of death (Figure 2). The three cases of intracranial hemorrhage were suspected child abuse as shown in Figure 3. On the contrary, PMCT could not identify nine cases of sepsis established by clinical diagnoses; nevertheless the focus of infection (three cases of pneumonia) and occult cancer (one case of malignant lymphoma) could be inferred from PMCT imaging. One case with an unknown cause of death at clinical diagnosis was attributed to mitochondrial myopathy at autopsy, which was misdiagnosed as pulmonary atelectasis by PMCT. One case of pneumothorax revealed by PMCT imaging was not detected at autopsy.

## 4. Discussion

In the present study, the percentage agreement on the cause of nontraumatic death in infants and children made by clinical diagnosis and by PMCT was 42% and that for the cause made by PMCT and by conventional autopsy was 57%. To our knowledge, this is the first study in Japan investigating PMCT in nonforensic cases of infants and children who suffered a nontraumatic death. Our findings agreed well with the previous report that whole-body PMCT might detect relevant findings that can help explain sudden unexpected death and detect nonaccidental injuries (abuse) in infants and children [9] although it is postulated that PMCT alone might not be sufficient to define the cause of death [10].

One of the main purposes of postmortem imaging is to detect child abuse. In our study, three cases of child abuse with central nervous system injury were found on PMCT although the clinical diagnosis was “unknown cause of death.” One of these cases was complicated by clavicular fracture. These findings suggest that postmortem imaging seems to play an important role in the detection of child abuse as it is difficult to detect child abuse only by surface inspection and history taking: PMCT provides important additional information regarding the trauma that may not be documented at autopsy. In fact, postmortem imaging including PMCT appears to be superior to autopsy in detecting fractures, as conventional autopsy does not routinely examine the whole skeleton [10]. Based on these findings, we recommend the routine use of PMCT in not only cases of suspected child abuse but all nontraumatic deaths in children.

The advantages of PMCT imaging include the detection of the following: good visualization of air distribution within the body (e.g., pneumothorax) and the location of foreign objects (e.g., catheters or drains) [8]. In agreement with these reports, one case of pneumothorax in the present study was misdiagnosed as pulmonary hypoplasia by conventional autopsy, and one case of cardiac tamponade was not detected as the cause of death by clinical diagnosis. On the contrary, there are some disadvantages of PMCT imaging. One of the most common major discrepancies between the autopsy- and radiology-derived cause of death was in the diagnosis of pneumonia [5]: radiologists interpreted the pneumonic consolidation as pulmonary edema secondary to cardiac failure; also, compared with immediate PMCT, delayed PMCT showed advanced dependent opacity and consolidation corresponding to congestive pulmonary edema. PMCT images of the lung change and the natural postmortem changes, such as pulmonary congestion and edema, begin to emerge as the time after death passes [11]. In fact, in the current study, PMCT could not differentiate antemortem pulmonary lesions due to other causes, that is, cardiac failure, aspiration pneumonia, or infectious pneumonia from postmortem changes including pulmonary congestion, pulmonary edema, or pulmonary atelectasis. Nonetheless, there is no doubt that PMCT imaging is useful as complementary information to identify the nontraumatic death more precisely in infants and children.

Our study has some limitations. First, the sample size of subjects who underwent conventional autopsy was small. A larger-scale prospective study should be conducted to establish the accuracy of postmortem imaging compared with conventional autopsy as the gold standard postmortem examination. Second, the two radiologists who interpreted PMCT images were general radiologists who had no previous experience of postmortem imaging. Third, we could not assess the interobserver variation in the radiologically diagnosed causes of death.

In summary, PMCT may be useful for identifying the cause of death in infants and children that traditionally have been identified by conventional autopsy. Particularly, it may help us to diagnose the child abuse in which the parents tend to refuse conventional autopsy. Larger multicenter trials are worthwhile to test the general feasibility of postmortem imaging, which appears to be a promising technique that might serve as a substitute for conventional medical autopsy, especially with the confirmation of clinical diagnoses.

## Abbreviations

CT: Computed tomography
MRI: Magnetic resonance imaging
PMCT: Postmortem computed tomography.

## References

[1] Burton JL, Underwood J (2007). Clinical, educational, and epidemiological value of autopsy. *The Lancet*.

[2] Brodlie M, Laing IA, Keeling JW, McKenzie KJ (2002). Ten years of neonatal autopsies in tertiary referral centre: retrospective study. *British Medical Journal*.

[3] Newton D, Coffin CM, Clark EB, Lowichik A (2004). How the pediatric autopsy yields valuable information in a vertically integrated health care system. *Archives of Pathology and Laboratory Medicine*.

[4] Kono T (2012). Post-mortem imaging in children. *Journal of the Japan Pediatric Society*.

[5] Roberts IS, Benamore RE, Benbow EW (2012). Post-mortem imaging as an alternative to autopsy in the diagnosis of adult deaths: a validation study. *The Lancet*.

[6] Takahashi N, Higuchi T, Shiotani M (2012). The effectiveness of postmortem multidetector computed tomography in the detection of fatal findings related to cause of non-traumatic death in the emergency department. *European Radiology*.

[7] Le Blanc-Louvry I, Thureau S, Duval C (2013). Post-mortem computed tomography compared to forensic autopsy findings: a French experience. *European Radiology*.

[8] Wichmann D, Obbelode F, Vogel H (2012). Virtual autopsy as an alternative to traditional medical autopsy in the intensive care unit; A prospective cohort study. *Annals of Internal Medicine*.

[9] Proisy M, Marchand AJ, Loget P (2012). Whole-body post-mortem computed tomography compared with autopsy in the investigation of unexpected death in infants and children. *European Radiology*.

[10] Oyake Y, Aoki T, Shiotani S (2006). Postmortem computed tomography for detecting causes of sudden death in infants and children: retrospective review of cases. *Radiation Medicine*.

[11] Shiotani S, Kobayashi T, Hayakawa H, Kikuchi K, Kohno M (2011). Postmortem pulmonary edema: a comparison between immediate and delayed postmortem computed tomography. *Legal Medicine*.

